# Tubercle Bacilli in the Fæces in Tuberculosis

**Published:** 1910-10-22

**Authors:** 


### PATHOLOGY.

### TUBERCLE BACILLI IN THE FÆCES IN TUBERCULOSIS.

The question of the extent to which tubercle bacilli occur in the fæces in cases of tuberculosis has recently been extensively investigated by Dr. R. W. Philip and Dr. Agnes Porter, and they have come to the conclusion that the bacilli are to be found in the fæces in cases of phthisis even more constantly than they are to be detected in the sputum; they appear to be quite convinced that as a means of diagnosis this method of examination is of great importance. They state with certainty that the occurrence of tubercle bacilli in the fæces is independent of the presence of any intestinal lesion. Of a hundred tuberculous cases examined, seventy-nine were found to yield tubercle bacilli, and only twenty-one were negative. Nine specimens from presumably normal subjects yielded negative results when examined in the same way. Of the patients known to be tuberculous all except one presented tubercle bacilli in the fæces, and as a strong indication of the value of this clinical method in certain instances it may be mentioned that of forty-two cases in which bacillary examination of the sputum was negative, twenty-nine exhibited tubercle bacilli in the fæces, whilst in twenty-four other cases in which there was no sputum obtainable for examination at all, seventeen showed the bacilli in the stools.

The method employed is that which may be termed the antiformin process of Uhlenhuth and Xylander. Antiformin consists of a mixture of an alkali hypochlorite and alkali hydrate. Acid-fast bacteria can resist this disinfectant when diluted to 20 per cent. for two to five hours, while other bacteria and organic matter are speedily dissolved. A small piece of fæces, about a cubic half-inch in size, is placed in a conical glass, and to this some 20 c.cm. of antiformin which has been diluted with water to 15 per cent. is added. The fæcal matter is well broken up and mixed with the fluid, and thereafter as much again of the dilute antiformin solution is added. This is further mixed and allowed to stand for about an hour. A white curdy precipitate appears on mixing, and in the course of an hour settles into a white layer which varies in breadth. Beneath the white layer some unchanged fæcal matter remains, and above it the fluid is of a clear yellow or brownish colour. A residue, including tubercle bacilli which have been set free by the destruction of the other constituents of the fæces, appears to be gathered into the white layer by a process of sedimentation. During the precipitation and sedimentation of the organic matter the bacteria are carried down, perhaps in some degree agglutinated. This concentration of the organisms makes up for the disadvantage caused by the initial dilution of the material.

For microscopic examination a drop is then taken from the white curdy layer by means of a pipette and placed at the end of a slide with a drop of albuminous water from a mixture containing one part of egg albumin to ten of distilled water and 1 per cent. of formalin, and 90 per cent. of distilled water is added. A film is made in the usual way by drawing out the fluid with a second slide along the surface of the first. The albumin, which is recommended by Seeman, helps to produce a better film and prevents it from being washed off.

As to the strength of antiformin to be used, 15 to 20 per cent. seems to be the most suitable. The use of a stronger solution, such as 50 per cent., is apt to lead to separation of the film. The time during which the action of the antiformin may be continued does not require very strict limitation; although Seeman says the bacilli cannot stand its action for twelve hours, it has not been possible to determine any deterioration of staining properties of the bacilli after twenty-four hours' action when use is made of the 15 to 20 per cent. dilution.

